# Stem cells as potential therapeutics for hearing loss

**DOI:** 10.3389/fnins.2023.1259889

**Published:** 2023-09-07

**Authors:** Qiaojun Fang, Yongjie Wei, Yuhua Zhang, Wei Cao, Lin Yan, Mengdie Kong, Yongjun Zhu, Yan Xu, Lingna Guo, Lei Zhang, Weiqing Wang, Yafeng Yu, Jingwu Sun, Jianming Yang

**Affiliations:** ^1^Department of Otolaryngology-Head and Neck Surgery, the Second Affiliated Hospital of Anhui Medical University, Hefei, Anhui, China; ^2^School of Life Sciences and Technology, Southeast University, Nanjing, China; ^3^Department of Otolaryngology-Head and Neck Surgery, The First Affiliated Hospital of Soochow University, Suzhou, Jiangsu, China; ^4^Department of Otolaryngology-Head and Neck Surgery, The First Affiliated Hospital of USTC, Division of Life Sciences and Medicine, University of Science and Technology of China, Hefei, Anhui, China

**Keywords:** hearing loss, stem cells, exosomes, nanomaterials, clinical trial

## Abstract

Hearing impairment is a global health problem. Stem cell therapy has become a cutting-edge approach to tissue regeneration. In this review, the recent advances in stem cell therapy for hearing loss have been discussed. Nanomaterials can modulate the stem cell microenvironment to augment the therapeutic effects further. The potential of combining nanomaterials with stem cells for repairing and regenerating damaged inner ear hair cells (HCs) and spiral ganglion neurons (SGNs) has also been discussed. Stem cell-derived exosomes can contribute to the repair and regeneration of damaged tissue, and the research progress on exosome-based hearing loss treatment has been summarized as well. Despite stem cell therapy’s technical and practical limitations, the findings reported so far are promising and warrant further investigation for eventual clinical translation.

## Introduction

Hearing impairment is one of the most prevalent sensory disorders worldwide, affecting millions. Hearing aids and cochlear implants cannot restore normal hearing, warranting new therapeutic approaches (Lieu et al., 2020). Stem cell therapy has gained considerable attention over the years due to its substantial regenerative potential.

Depending on the location of the damage in the auditory system, deafness is divided into conductive and sensorineural types (Seddon et al., 2012). Conductive deafness occurs due to lesions in the tympanic membrane and the auditory tuberosity, which impede sound transmission to the inner ear (Lauer et al., 2019). On the other hand, sensorineural deafness is mainly the result of lesions in the auditory center, including the inner ear and the auditory nerve. HCs and SGNs are crucial in transmitting peripheral acoustic signals (Nayagam et al., 2011; Moser and Starr, 2016). However, mammalian cochlear HCs do not regenerate spontaneously after injury (Swan et al., 2008; Omichi et al., 2019). Causes of sensorineural deafness include noise, aging, drug cause hearing loss, genetics, bacterial and viral infections, immunological diseases, and endolymph fluid (Meniere’s disease) (Wang and Puel, 2018; Plontke et al., 2022). Currently, induction of stem cell differentiation and replacement of damaged HCs and SGNs are increasingly considered feasible treatment options for auditory regeneration.

### Historical overview of stem cell research

**Figure 1:**
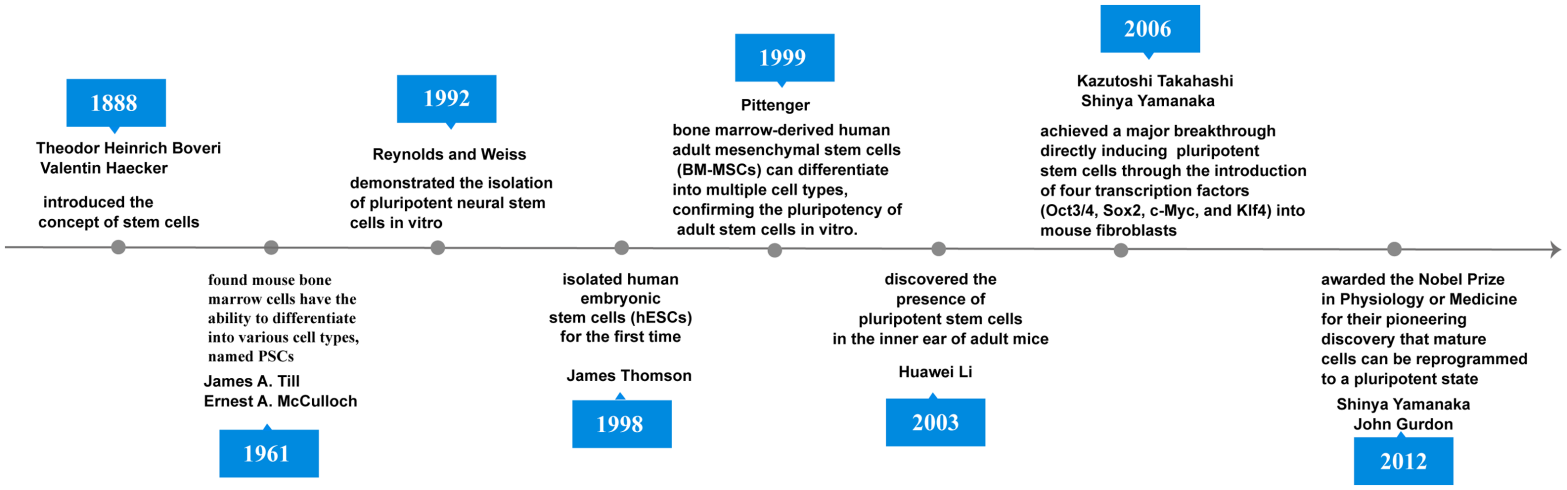
The timeline of major discoveries and breakthroughs in stem cell research.

The origin of stem cell therapy dates back to 1888. when German zoologists Theodor Heinrich Boveri and Valentin Haecker introduced the concept of stem cells, they identified various cell populations in the embryo that could differentiate into specific cell types (Ramalho-Santos and Willenbring, 2007). In 1961, Till and Mc (1961) discovered that stem cells obtained from mouse bone marrow cells, which could differentiate into various cell types, and termed pluripotent stem cells (PSCs). Reynolds and Weiss (1992) isolated pluripotent neural stem cells (NSCs) from the forebrain of adult mammals in 1992. Thomson et al. (1998) first isolated human embryonic stem cells (hESCs) from embryos in 1998. In 1999, Pittenger et al. showed that bone marrow-derived human adult mesenchymal stem cells (BM-MSCs) can differentiate into multiple cell types, thus demonstrating the pluripotency of adult stem cells (ASCs) *in vitro*. BM-MSCs exist in almost all tissues and are crucial for maintaining tissue homeostasis through their self-renewal capacity (Pittenger et al., 1999; Tuan et al., 2003). Huawei et al. identified PSCs in the inner ear of adult mice and found that these cells could self-renew and differentiate into HC-like cells (HCLs) when cultured *in vitro* 16 (Li et al., 2003a). Takahashi and Yamanaka used the four transcription factors Oct3/4, Sox2, c-Myc, and KLF4 to transform mouse fibroblasts into induced pluripotent stem cells (iPSCs) for the first time (Takahashi and Yamanaka, 2006; Takahashi et al., 2007). This groundbreaking 2006 study paved the way for reprogramming mature somatic cells into a pluripotent state and opened new avenues for stem cell research. For this discovery, Shinya Yamanaka and John Gurdon received the Nobel Prize in Physiology or Medicine in 2012 (Figure 1) (Johnson and Cohen, 2012). Over the past decade, stem cell-based therapies have garnered considerable attention in hearing loss treatment.

### The diversity of stem cells in regenerative medicine

Stem cells are a group of undifferentiated cells that can self-renew and differentiate into one or more cell types at different times of life (Ho et al., 2012; Hao et al., 2020). Based on their origin, stem cells can be categorized into various types, such as embryonic stem cells (ESCs), iPSCs, adult or somatic stem cells, and NSCs (Bongso and Richards, 2004; Ilic and Polak, 2011; Bond et al., 2015).

ESCs are pluripotent stem cells derived from the inner cell mass of blastocysts formed 5–6 days after fertilization (Evans and Kaufman, 1981). All three ectoderm, mesoderm, and endoderm germ layers can be differentiated from ESCs (Yao et al., 2006). ESCs can be obtained by culturing inner cell masses isolated from trophoblasts under specific conditions (Bongso, 2006).

IPSCs are produced by reprogramming mature somatic cells into ESC-like cells through genetic or chemical intervention (Yamanaka, 2012; Hockemeyer and Jaenisch, 2016). IPSCs are suitable models for investigating disease treatment, drug discovery, and regenerative medicine because they can self-renew and differentiate into various cell types (Ohnuki and Takahashi, 2015). Somatic cells can be reprogrammed to iPSCs by transducing them with the Oct4, Sox2, Klf4, and c-Myc transcription factors (Takahashi et al., 2007). In addition, certain chemicals or microenvironmental factors have also been used to stimulate the generation of iPSCs.

Adult or somatic stem cells are undifferentiated cells derived from various adult tissues with pluripotency, self-renewal, and limited differentiation potential (Zakrzewski et al., 2019). MSCs are the most common adult or somatic stem cells. Among them, BM-MSCs have limited differentiation capacity for osteocytes, chondrocytes, and adipocytes (Caplan, 2010). Although their differentiation capacity is limited, they exhibit anti-inflammatory properties and augment tissue regeneration (Ilancheran et al., 2009; Moodley et al., 2010).

NSCs have remarkable self-renewal and differentiation capabilities and continuously generate new neurons and glial cells (Xing et al., 2021). They play crucial roles in embryonic development and post-natal growth, particularly in the brain and spinal cord, wherein they help maintain neural tissue homeostasis and regenerative capacity (Shao et al., 2019). NSCs are also the seed cells for neural stem cell therapy and can promote nerve regeneration and restore function when implanted into damaged nerve tissue (Wang et al., 2019). The clinical applicability of NSCs is constantly being explored for treating neurological diseases.

### Stem cell therapy for hearing impairment

**Figure 2:**
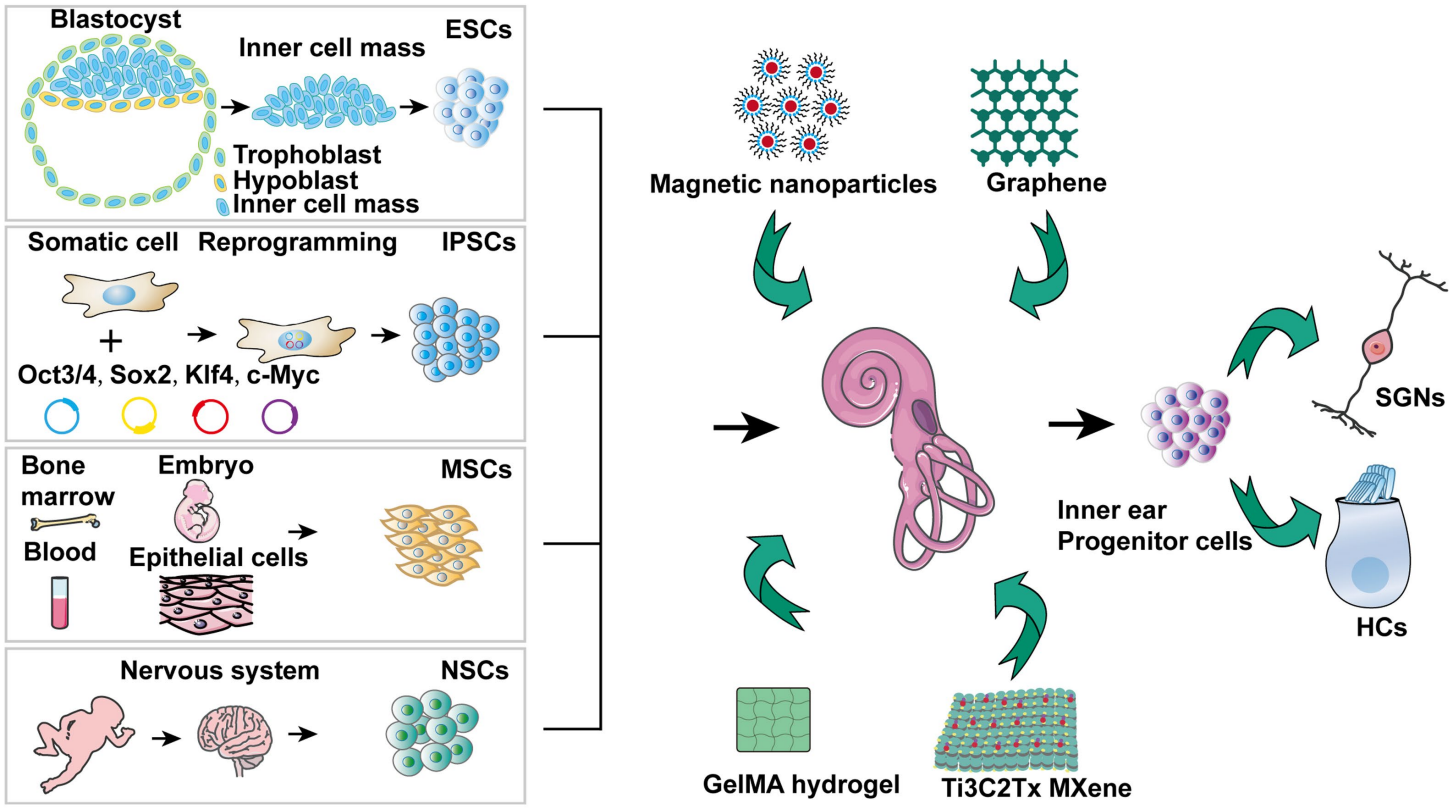
Stem cell treatment for hearing impairment mechanisms. 1) ESCs are able to differentiate into that resemble SGNs, SCs, and HCs, offering potential replacement strategies. 2) A particular strategy can be realized by stimulating iPSCs from patients who have hearing loss for developing into HCLs. 3) A range of growth factors and cytokines are secreted by MSCs, which may help prevent hearing loss. 4) NSCs together with nanomaterials hold promise for protecting against hearing loss. 5) The inner ear precursor cells are capable of being stimulated to develop into HCs and SGNs, offering another avenue for regeneration. 6) Exosomes derived from stem cells demonstrate potential in preventing sensorineural hearing loss.

Due to their capacity to differentiate into numerous cell types and repair tissues that have been damaged, stem cells may offer a promising treatment option for hearing loss (Bacakova et al., 2018; Camp et al., 2018). The ESCs, iPSCs, and ASCs have been tested for treating hearing impairment (Boer et al., 2009; Stojkovic et al., 2021; Zine et al., 2021). Nevertheless, each variety has advantages and disadvantages concerning differentiated future potential applicability and immunogenicity (Figure 2) (He et al., 2021). In hearing loss research, stem cells have successfully generated HCLs *in vitro* (Li et al., 2003b; Takeda et al., 2018).

### ESCs for treating hearing loss

Recent studies have shown that hESCs can be differentiated *in vitro* into cochlear sensory epithelial cells containing HCs using a three-dimensional culture system (Koehler et al., 2013). In addition, hESCs have also been differentiated into purified ear nerve precursor cells and spiral ganglion-like cells, which can survive for extended periods *in vitro* (Matsuoka et al., 2017). hESC-derived precursor cells transplanted into the cochlear region of Pou4f3DTR/+ mice with selective diphtheria toxin-induced HC ablation were viable and differentiated into HC-like and SC-like cells (Takeda et al., 2021). These findings suggest hESCs may be a potential treatment for hearing impairment and warrant further investigation.

### IPSCs for the treatment of deafness

Recently, iPSCs have become known as a potential biological treatment for deafness. IPSCs derived from human urinary cells obtained from donors in good health were differentiated into HCLs with the morphological and electrophysiological characteristics of inner ear HCs. These HCLs established synaptic connections with the SGNs that were co-cultured. In addition, the transplanted iPSCs migrated to the organ of Corti site of resident HCs, differentiated into HCLs, and established synaptic links with the native SGNs (Chen J. et al., 2018). Somatic cells from patients with *myosin7a* and *myosin15a* mutations were also reprogrammed into iPSCs, and the mutations were corrected using gene editing techniques. Restoring gene function in iPSCs enabled differentiated HCLs to regain morphology and functioning (Chen et al., 2016; Tang et al., 2016). Similarly, iPSCs derived from patients with A8344G and *trmu* mutations in mitochondrial DNA were differentiated into inner ear HCLs. These cells exhibited normal electrophysiological properties after gene restoration (Chen Y. C. et al., 2018; Chen and Guan, 2022). These cellular models can elucidate the functional connection between inner ear HCs development and mitochondrial DNA. Additionally, human iPSCs derived from skin cells of patients with connexin 26 mutations, encoded by GJB2, were differentiated into auditory neural progenitor and hair cell precursor cells (Fukunaga et al., 2021). Connexin 26 mutations are a common cause of hereditary deafness. Overall, these findings provide novel insights and highlight potential therapeutic uses of iPSCs for treating hearing loss.

### The therapeutic potential of MSCs for hearing loss

Although ESCs and iPSCs can differentiate into inner ear HCs, their application in medicine is limited due to the risk of tumorigenicity. Direct reprogramming of fibroblasts into HCLs of the inner ear could be a viable alternative. Mouse Embryonic Fibroblasts can be differentiated into HCLs *via* mesenchymal-to-epithelial transition, followed by increased the expression of three important transcription factors, Sox2, Eya1 and Six1, to induce ear-sensory epithelial cell characteristics (Yang et al., 2021). Conductive hearing loss is commonly caused by cerumen embolism and chronic otitis media, leading to perforation of the tympanic membrane and erosion of the auditory ossicles due to recurrent infections. While the tympanic membrane can be regenerated using fascia or perichondrium, stem cells are essential for effectively enhancing hearing (Goncalves et al., 2017; Maharajan et al., 2020). In the rat model of subacute tympanic membrane perforation, bioprinted polycaprolactone/collagen/alginate-mesenchymal stem cell scaffolds have demonstrated efficacy and feasibility for subacute tympanic membrane regeneration (Jang et al., 2017). BM-MSCs have also been shown to promote healing in a chronic tympanic membrane perforation rat model (Shahal et al., 2022). Other ossicles or cartilage may be utilized to surgery restore hearing in cases of bone erosion. Additionally, MSCs have demonstrated promise in the therapy of conductive hearing loss (Maharajan et al., 2020). The resident MSCs protect the cochlear epithelium and prevent noise-induced hearing damage by secreting various growth factors and cytokines (Warnecke et al., 2021a). Moreover, pre-treatment of MSCs with deferoxamine can enhance their homing ability, which refers to the migration ability to damaged sites, through activation of the PI3K/AKT signaling pathway (Peyvandi et al., 2018).

### NSCs for hearing loss

There have been considerable efforts in recent years to treat sensorineural hearing loss by inducing the regeneration of damaged auditory HCs and SGNs (Wang and Puel, 2018; Shu et al., 2019). The combination of nanomaterials and stem cells is a promising new therapeutic approach against hearing loss that combines the proliferation capacity of the stem cells with the tissue-targeting ability of the nanocarriers (Chang et al., 2020; Zhang et al., 2022). Several studies have demonstrated that stem cells and nanomaterials can support auditory regeneration by accelerating the repairing of damaged tissues (Zhong et al., 2016; Hu et al., 2021).

Graphene, a single layer of carbon atoms arranged in a hexagonal lattice, has been shown to play a critical role in tissue reconstruction (Kim et al., 2013; Akhavan, 2016; Guo et al., 2016). Autologous tissue grafts of perforated tympanic membranes can restore low-frequency hearing but often impair high-frequency hearing. In a rat deafness model, thin multilayer graphene membranes restored broadband hearing by inducing tympanic membrane repair (Li C. et al., 2022). In addition, the electrical stimulation device was developed by the combination of a cochlear implant and NSCs cultured on a graphene substrate. The machine was biocompatible and induced regeneration of NSCs in response to high-frequency, high-amplitude electroacoustic stimulation (Guo et al., 2021).

Magnetic nanoparticles are widely used in biomedical applications such as magnetic labeling, magnetic imaging, tumor treatment, and drug delivery due to their good biocompatibility (Lin et al., 2021; de Vincentiis et al., 2023). Superparamagnetic iron oxide nanoparticles can promote the proliferation of NSCs in a static magnetic field by enhancing cell cycle progression (Li et al., 2021). Furthermore, the directed growth of cochlear spiral neurons can be regulated by magnetic field-induced self-assembly of magnetic nanoparticles into multi-directional nanowires (Xia et al., 2022).

GelMA hydrogel is synthesized from methacrylic anhydride (MA) and gelatin. It is an ideal scaffold for 3D cell culture, tissue engineering, and biological 3D printing due to its excellent biocompatibility and visible light-curing properties (Fan et al., 2018; Cai C. et al., 2022). Composite scaffolds of super-aligned carbon nanotubes and GelMA promote the SGNs growth and orientation (Hu et al., 2022). Grooved GelMA-MXene enhanced the adhesion, differentiation, and directed proliferation of NSCs *in vitro* (Cai J. et al., 2022). Ti3C2Tx MXene, composed of transition metals, carbides, nitrides, or carbonitrides, exhibits a large surface area, adjustable surface functional groups, and good electrical conductivity (Rasool et al., 2016; Wu et al., 2021; Serles et al., 2022). It can enhance the proliferation and neural differentiation of NSCs, and promote the development of SGN growth cones and neurite growth by delivering electrical stimuli (Guo et al., 2022; Liao et al., 2022; Li Y. et al., 2022).

### Inner ear progenitors for auditory regeneration

HCs and supporting cells (SCs) are critical inner ear components that arise from a common sensory progenitor. Inner ear progenitor cells are pluripotent cells with self-renewal ability that can differentiate into HCs under suitable induction conditions. During embryonic development, signaling pathway regulation plays vital roles in the formation of the organ of Corti. Activation of the Wnt pathway and inhibition of the Notch pathway promote partial regeneration of HCs (Mizutari et al., 2013; Shi et al., 2014; Li et al., 2015). Lgr5, a receptor of the Wnt pathway, is also a marker of cochlear stem cells (Chai et al., 2012; Shi et al., 2013; Bramhall et al., 2014). Under specific conditions, Lgr5-expressing Sertoli cells can transdifferentiate into HCs postnatally (McLean et al., 2017). Additionally, Sox2 is crucial for cell division and differentiation during development. Inner ear epithelial cells of Sox2 haploinsufficient mice showed increased differentiation and proliferation, resulting in expanded HCs and SCs and eventual regeneration of cochlear function. Sox2 haploinsufficiency also activates the cochlear Wnt pathway, further enhancing regeneration (Atkinson et al., 2018; Steevens et al., 2019).

The let-7 microRNA is a conserved activator that promotes proliferative quiescence and terminal differentiation by repressing CHD7, which controls progenitor cell behavior during cochlear development. Inhibition of let-7 in chicken auditory organ slices prolonged pre-sensory cell differentiation and proliferation (Evsen et al., 2020; Nie et al., 2022). In mice, the RNA-binding protein LIN28B promotes HC generation from auditory SCs *via* the mTOR pathway during embryonic development (Li and Doetzlhofer, 2020). The Yap-Lin28a axis can also activate Wnt signaling and promote inner ear cell regeneration by inhibiting let-7 expression (Kempfle et al., 2020; Ye et al., 2020). Knockdown of Foxg1 in neonatal mouse SCs promoted their transdifferentiation into HCs (Zhang et al., 2020). Furthermore, the Yap/Tead complex regulates a proliferation gene network in cochlear progenitors. Tead transcription factors directly bind regulatory elements of stem cell and cell cycle genes. In Sox2-positive cells, Yap as a Tead activator is rapidly degraded (Gnedeva et al., 2020; Currey et al., 2021). The transcriptional repressors TBX2 and TBX3 play essential roles in cochlear morphogenesis (García-Añoveros et al., 2022; Kaiser et al., 2022). Loss of Tbx2 causes cochlear hypoplasia, while Tbx3 mutants exhibit inner ear morphogenesis defects (Vitelli et al., 2003; Kaiser et al., 2021; Bi et al., 2022; Kaiser et al., 2022). The transcription factor ATOH1 promotes HC differentiation by upregulating Pou4f3, which facilitates ATOH1 binding and activation of other target genes (Yu et al., 2021; Costa et al., 2022). As a transcriptional activator of Sonic hedgehog (Shh), Gli2 is negatively regulated by Suppressor of Fused Homolog (Sufu). Controlling Gli2 is critical for regulating cochlear HC differentiation, as Sufu inhibition can disrupt Atoh1 expression and delay differentiation (Yin et al., 2019; Qin et al., 2022). Overexpression of Rps14 in the mouse cochlea promotes SC proliferation by activating Wnt signaling and inducing HC regeneration (Xu et al., 2023). These studies show that co-regulation of the Wnt, Notch and Shh pathways promotes HCs regeneration, and provides a new insight for the potential application of HC regeneration.

### Stem cell-derived exosomes have broad therapeutic prospects in hearing impairment

**Figure 3:**
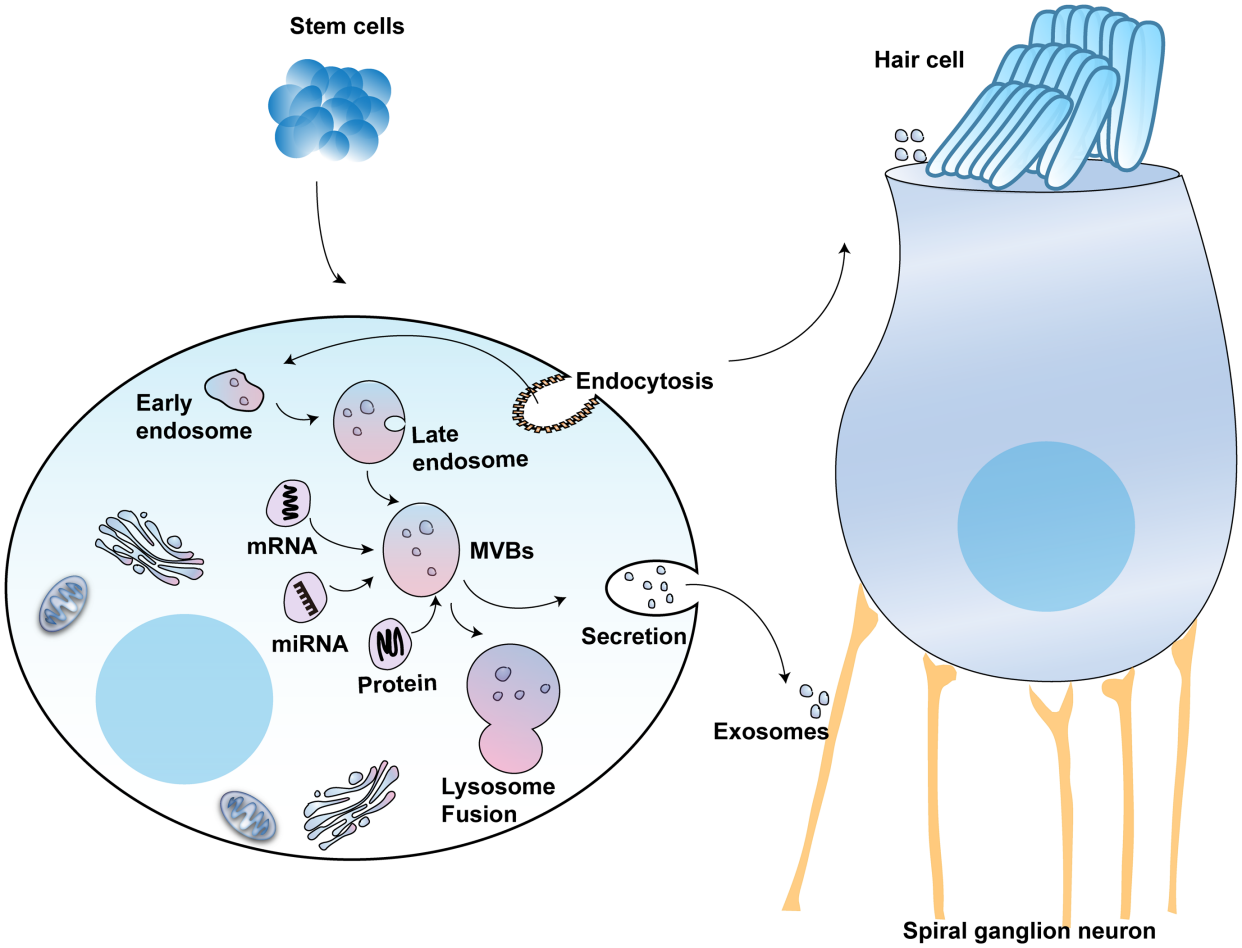
The potential use of exosomes in hearing protection: The schematic model shows the molecular composition of exosomes formed from stem cells, which include a wide range of cargo molecules, such as DNAs, RNAs, and proteins. Exosomes develop when the lipid bilayer membrane of MVBs undergoes an inward budding process. These MVBs can either be degraded by lysosomes or combined with the plasma membrane, releasing exosomes.

A class of small extracellular vesicles called exosomes that diameters ranging between 30 and 150 nm (Kalluri and LeBleu, 2020). Many cells, such as immune cells, cancer cells, and stem cells, can secrete exosomes (Yang et al., 2019; Cully, 2021). Exosomes derived from various cell types are highly heterogeneous. However, stem cell-derived exosomes have multiple mechanisms for repairing tissue damage, including promotion of cell proliferation and survival, enhancement of angiogenesis, and inhibition of inflammation and oxidation. For example, exosomes secreted by adipose-derived mesenchymal stem cells that are enriched in miR-25-3p induced neuroprotection through activation of autophagic flux (Kuang et al., 2020). The formation of exosomes through the endocytic pathway includes the following process: cytoplasmic membrane invagination, encapsulating some extracellular components and cell membrane proteins to form early endosomes (ESEs), followed by fusion between different ESEs to form late endosomes (LSEs), and further formation of multivesicular bodies (MVBs) (Chang et al., 2021). MVBs contain many intraluminal vesicles (ILVs) that may be released into exosomes (Han et al., 2022). MVBs are degraded by fusion with lysosomes or by fusing with the plasma membrane, releasing their substances, including ILVs, which are the final exosomes (Figure 3) (Kumar et al., 2020).

Exosomes deliver the vesicle’s load, such as lipids, proteins, and other molecules, to the destination cells (Sun et al., 2020). Studies show that these exosomes can promote the regeneration of damaged tissues, modulate cellular immune responses, and reduce cellular inflammatory responses by activating specific signaling pathways (Dai et al., 2020; Ocansey et al., 2020; Xu et al., 2020; Cao et al., 2021; Isaac et al., 2021). Exosomes derived from stem cells can help regenerate neurons and synapses, alleviating the symptoms of neurodegenerative disorders (Vogel et al., 2018; Riazifar et al., 2019; Guo et al., 2020; Fayazi et al., 2021). Moreover, exosomes play an important role in cochlear sensory HCs protection. After stress stimulation, the cochlear SCs can release exosomes containing heat shock protein 70 (HSP70). To prevent the death of HCs, HSP70 takes a paracrine method to act on toll-like receptor 4 (TLR4) (Breglio et al., 2020; Muller, 2020). Another study showed that extracellular vesicles from human vestibular schwannomas are able to damage cochlear HCs and SGNs, leading to hearing loss (Soares et al., 2016).

Exosomes have also been demonstrated to protect against drug-induced hearing loss. For example, in response to cisplatin and other drugs *via* the HSP70 pathway, exosomes secreted by BM-MSCs reduced the apoptosis of mouse cochlear HCs (Park et al., 2021). Furthermore, human MSCs were able to regenerate SGNs and restore hearing in mice with autoimmune sensorineural deafness induced by β-tubulin through paracrine activity (Tsai et al., 2021). Furthermore, human MSCs were able to regenerate SGNs and restore hearing in mice with autoimmune sensorineural deafness induced by β-tubulin through paracrine activity (Yoo et al., 2015). Human MSC-derived extracellular vesicles also protect against noise-induced deafness in mice (Warnecke et al., 2020; Huang et al., 2023).

## Clinical trials of stem cell therapy for deafness

Although multiple cellular and animal studies have demonstrated the security and feasibility of stem cell treatment for deafness, stem cell-based clinical trials for deafness treatment are still scarce. Alpha mannosidase deficiency is a rare genetic disorder that can lead to multi-organ dysfunction and cognitive deficits. One clinical study showed that five patients with α-mannosidase deficiency significantly improved their symptoms after transplantation of the allogeneic hematopoietic stem cells (Grewal et al., 2004). Blood cells, nerve cells, and cardiomyocytes can differentiate from umbilical cord stem cells. Studies have shown that after transplanting stem cells from autologous cord blood, auditory function is restored in children with acquired sensorineural hearing loss (Baumgartner et al., 2018; Sun and Yang, 2020). miR-22-3p, a microRNA relatively highly expressed in mesenchymal stem cell-derived exosomes, reduces inflammation by inhibiting expression of NLRP3. Additionally, mesenchymal stem cell-derived exosomes significantly inhibit expression of the pro-inflammatory factors TNF-α, IL-1β, and iNOS while promoting expression of the anti-inflammatory factor IL-10, thereby suppressing inflammation (Liu et al., 2020; Wang et al., 2023). In a clinical trial concluded in 2021, human umbilical cord MSCs-derived extracellular capsules were transplanted into the inner ear, reducing the inflammatory side effects caused by cochlear implantation (Warnecke et al., 2021b).

## Conclusion

This review discusses the present status of the use of MSCs, ESCs, iPSCs, inner ear progenitor cells, and NSCs in the repair and regeneration of auditory impairment. MSCs are easily accessible and expandable and are, therefore, the most commonly used stem cell type. ESCs and iPSCs have strong differentiation potential, but their clinical application is limited due to ethical and safety concerns. Cells that can differentiate into cochlear HC and spiral neurons are inner ear progenitor cells, a type of ASCs. Although several preclinical and clinical studies have proved the therapeutic potential of stem cells in auditory impairment, Stem cell therapy also has some significant limitations, such as safety and feasibility. Specifically speaking, stem cell transplantation carries risk of tumourigenesis and immune rejection after transplantation, and existing delivery methods for stem cells can affect their therapeutic efficiency. In addition, the ethical issues also need to be addressed. In the future, the source of stem cells and the time and cell dosage for treatment will be optimized, More and more superior biomaterials and targeted delivery modalities will be developed. Overall, stem cell therapy is a brilliant way to restore hearing loss.

## Author contributions

QF: Conceptualization, Writing – original draft. YW: Data curation, Writing – original draft. YuZ: Formal analysis, Writing – original draft, Data curation. WC: Resources, Writing – original draft, Formal analysis. LY: Writing – original draft, Funding acquisition. MK: Writing – original draft, Investigation. YoZ: Formal analysis, Writing – original draft, Methodology. YX: Writing – original draft, Project administration. LG: Writing – original draft, Resources. LZ: Project administration, Resources, Writing – original draft, Software. WW: Writing – original draft, Supervision. YY: Funding acquisition, Supervision, Writing – review & editing. JS: Funding acquisition, Validation, Writing – review & editing, Supervision. JY: Funding acquisition, Supervision, Writing – review & editing, Writing – original draft, Validation.

## Funding

The author(s) declare financial support was received for the research, authorship, and/or publication of this article. The writing of this manuscript was supported by the National Natural Science Foundation of China (82071055, 82271178, and 82271162), the Anhui Province Scientific Research Preparation Plan Project (2022AH050685, 2022AH050655), the Anhui Natural Science Foundation of China (2208085MH223), the Second Affiliated Hospital of Anhui Medical University National Natural Science Foundation Incubation Program (2022GMFY04), Anhui Province Key Specialties Construction Project in Medical and Health Care, the Project of State Key Laboratory of Radiation Medicine and Protection, Soochow University (GZK 1202209).

## Conflict of interest

The authors declare that the research was conducted in the absence of any commercial or financial relationships that could be construed as a potential conflict of interest.

## Publisher’s note

All claims expressed in this article are solely those of the authors and do not necessarily represent those of their affiliated organizations, or those of the publisher, the editors and the reviewers. Any product that may be evaluated in this article, or claim that may be made by its manufacturer, is not guaranteed or endorsed by the publisher.
